# Design and High Expression of Non-glycosylated Lysostaphins in *Pichia pastoris* and Their Pharmacodynamic Study

**DOI:** 10.3389/fmicb.2021.637662

**Published:** 2021-03-18

**Authors:** Wenluan Shen, Na Yang, Da Teng, Ya Hao, Xuanxuan Ma, Ruoyu Mao, Jianhua Wang

**Affiliations:** ^1^Gene Engineering Laboratory, Feed Research Institute, Chinese Academy of Agricultural Sciences, Beijing, China; ^2^Key Laboratory of Feed Biotechnology, Ministry of Agriculture and Rural Affairs, Beijing, China

**Keywords:** lysostaphins, non-glycosylation, *P. pastoris* expression, sepsis mouse model, pharmacodynamics

## Abstract

Lysostaphin is an effective antimicrobial agent to *Staphylococcus*, especially for the methicillin-resistant *Staphylococcus aureus* (MRSA) and multidrug-resistant *Staphylococcus aureus* (MDRSA). In this study, the seven lysostaphin derived mutants (rLys) were designed to overcome the barrier of glycosylation during expression in *Pichia pastoris*. Among them, 127A and 127A232Q had highest antimicrobial activity (MIC values 0.07–0.3 μM) to *S. aureus* than others and the commercial lysostaphins (1–15.8 times). There was no glycosylation during the expression in 5-L fermenter level, with the high yield of 1315 mg/L (127A) and 1141 mg/L (127A232Q), respectively. Meanwhile, 127A and 127A232Q effectively killed 99.9% of *S. aureus* at low concentration (1 × MIC) within 30 min, without the regrowth of pathogen. They also showed low toxicity, high pH and temperature stability. The results of *in vivo* therapeutic effect of 127A and 127A232Q against high virulent *S. aureus* CVCC546 showed that 127A and 127A232Q increased the survival rate of infected mice up to 100% at the dose of 10 mg/kg than the untreated group, reduced the bacterial translocation by 5-7 log CFU (over 99%) in organs compared to the untreated group and alleviated multiple-organ injuries (liver, kidney and spleen). These data indicated that the non-glycosylated lysostaphin 127A and 127A232Q may be a promising therapeutic agent against MDR staphylococcal infections.

## Introduction

Due to the chronic overuse of antibiotics, many resistant *Staphylococcus aureus* (*S. aureus*) emerged in recent years, such as methicillin-resistant *S. aureus* (MRSA), vancomycin-resistant *S. aureus* (VRSA), and multiple-drug-resistant *S. aureus* (MDRSA), this *S. aureus* become a formidable pathogen which can cause terrible infections in humans and livestock. Meanwhile, between 2 and 53 million persons are estimated to carry MRSA worldwide (Gleeson et al., 2015). In the United States, the fees for the 6-month treatment regimen against MRSA infection amount to nearly $36,000 in 2013 (Laxminarayan et al., 2013). It has been estimated there would be about 10 million deaths every year and $100 trillion lost to the global economy by 2050 (Review on Antimicrobial Resistance, 2017^1^). Therefore, the new anti-*S. aureus* agents with potent activity and low resistance are urgent needed.

Lysostaphin is a zinc metalloprotease from *Staphylococcus staphylolyticus* (Schindler and Schuhardt, 1964), and it has the activity of glycylglycine endopeptidase to lyses other *Staphylococcus* species such as *S. aureus.* The minimum inhibitory concentrations (MICs) of lysostaphin to various strains of *S. aureus* were ranged from 0.001–8.0 μg/mL (Climo et al., 1998; von Eiff et al., 2003; Yang et al., 2007). As early as in 1974, a 500 mg single dose of lysostaphin could showed eutherapeutic efficacy in a myelocytic leukemia patient suffering from multidrug-resistant staphylococcal pneumonia, multiple metastatic staphylococcal abscesses, and cellulitis (Zygmunt and Tavormina, 1972; Stark et al., 1974). Lysostaphin is very effective in protecting mouse models of systemic *S. aureus* infection from both methicillin-sensitive *S. aureus* (MSSA) and MRSA (Kokai-Kun et al., 2003). In the murine model of catheter-associated *S. aureus* biofilms, systemic lysostaphin administration was shown to clear established biofilms during a 4-day treatment, and a single prophylactic dose prevented subsequent biofilm formation on indwelling cath (Kokai-Kun et al., 2009).

The gene of lysostaphin was cloned in 1987 (Recsei et al., 1987; Kokai-Kun, 2012). The preproenzyme of lysostaphin is composed of 493 amino acids. The first 36 amino acids at the N-terminal are signal peptides region. When protein trafficking, they are located in the endoplasmic reticulum and then removed. This full protein sequence with 457 amino acids consisted of a propeptide of 211 amino acids from which 195 amino acids are assembled in 15 tandem repeats of 13 amino acids length, and a hydrophobic mature lysostaphin 246 amino acids (27 KDa) (Bastos et al., 2010). The crystal structure of mature lysostaphin has been resolved (Sabala et al., 2014), and it mainly includes three parts: 132 amino acids of catalytic domain (CAT), 102 amino acids of cell-wall-targeting domain (CWT) and 13 amino acids of linker between them.

The mechanism of lysostaphin to bacteria is to destroy the cell wall of *Staphylococcus* (Johnson et al., 2018; Gonzalez-Delgado et al., 2020). Pentaglycine crossbridges of peptidoglycan (PG) is the component of *Staphylococcus aureus* cell wall and an explicit target of Lys (Vollmer et al., 2008). When Lys combined with PG, the CAT domain destroys the bond between the second and third Glycine, and the structure of the cell wall of *S. aureus* is damaged, achieving a sterilization effect (Tossavainen et al., 2018; Grishin et al., 2019).

The clinical application of lysostaphin depends on the availability of large amounts of highly purified protein from a safe and nonpathogenic source. Firstly, lysostaphin was extracted from the culture of its natural host *S. simulans* (Chandra et al., 2018). Due to the technical difficulty, and the safety dispute, it cannot be used in actual production (Kokai-Kun, 2012). At present, most of the lysostaphin is obtained from the recombined expression, among which prokaryotic expression systems include *E. coli* (Sharma et al., 2006; Duman et al., 2019), and *Lactococcus lactis* (Mierau et al., 2005), with the yield of 50–300 mg/L, meanwhile, they had the disadvantages of higher cost and complex purification process. The eukaryotic expression systems include *Pichia pastoris* (Zhao et al., 2014), animal mammary epithelial cells (Fan et al., 2002), and etc. With the yield of 200–1000 mg/L. In the eukaryotic expression system, the expression of lysostaphin has encountered the barrier of glycosylation (Chen and Harcum, 2006). It has been shown that the activity of these glycosylated lysostaphins is lower than that of the non-glycosylated enzymes, and the other band is not easy to separate during purification. The glycosylation sites mutant could disrupt the N-terminal glycosylation of lysostaphin in *P. pastoris*. However, the yield of the derived lysostaphin was 500 mg/L (Zhao et al., 2014), which still have gaps to the demand of large-scale industrial production. In this study, the glycosylation site of lysostaphin was modified to obtain the non-glycosylated lysostaphin mutants. The 127A and 127A232Q had highest activity and yields. And the *in vivo* and *in vitro* activities were characterized. Additionally, the efficacy of 127A and 127A232Q were determined in a peritonitis mouse model of lethal infection with MDR *S. aureus*.

## Materials and Methods

### Strains, Plasmids and Reagents

The *Escherichia coli* DH5α, *P. pastoris* X-33, and pPICZαA vectors used in cloning and expression were purchased from Invitrogen. The restriction enzymes were purchased from Tiangan Biotech (China, Beijing). The test strains, including *S. aureus* ATCC 43300, *S. aureus* ATCC 25923 were purchased from the American Type Culture Collection (ATCC) (Beijing, China), *S. aureus* CICC 10436, *S. aureus* CICC 10473, *S. aureus* CICC 21601, *S. epidermidis* CICC 23962, *S. epidermidis* CICC 10294 were purchased from China Center of Industrial Culture Collection (CICC) (Beijing, China), *S. aureus* CVCC 546 were purchased from China Veterinary Culture Collection Center(CVCC) (Beijing, China), *S. epidermidis* CGMCC 1.4206 was purchased from China General Microbiological Culture Collection Center (CGMCC) (Beijing, China), *S. hyicus* NCTC 10350 was purchased from National Collection of Type Cultures (NCTC). The bovine clinical MDR *S. aureus* E48 strain was donated by Prof. Zhao Xin (Northwest A&F University) and numbered as FRI-GEL160701. The *S. aureus* ky, *S. hyicus* 437-2, *S. sciuri* 26, *S. sciuri* 31 were obtained from clinical trials and numbered as FRI-GEL180901, ACCC61734 (Agricultural Culture Collection of China), FRI-GEL180902, FRI-GEL180903, respectively.

The enzymes used in DNA restriction and DNA linkage were purchased from New England Biolabs (NEB, Beijing, China). The plasmid and DNA extraction kits were purchased from TIANGEN Biotech (Beijing, China). The commercial Lysostaphin was purchased from Sigma-Aldrich (Shanghai, China). Other reagents were analytical grade.

### Design of Non-glycosylated Lysostaphin Genes

**FIGURE 1:**
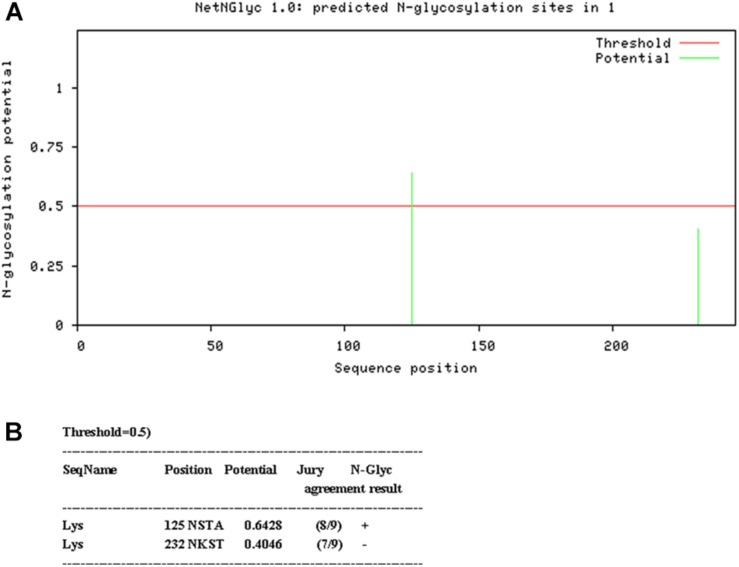
Analysis of glycosylation site of Lys sequence. **(A)** Probability of glycosylation at two potential sites **(B)** Result of Glycosylation site prediction.

N-linked glycosylation sequons were identified by NetNGlyc 1.0 Server^2^ (Figure 1). In order to disrupt the aberrant glycosylation of the wild-type protein sequence, seven optimized recombinant Lysostaphin (rLys) sequences were designed including 125Q (Asn to Gln at position 125), 232Q (Asn to Gln at position 232), 125Q232Q (Asn to Gln at positions of both 125 and 232), 126P (Ser to Pro at position 126), 126P 232Q (Ser to Pro at position 126 and Asn to Gln at position 232), 127A (Thr to Ala at position 127), 127A232Q (Thr to Ala at position 127 and Asn to Gln at position 232) and a wild-type lysostaphin (Lys) sequence. Codon optimized gene sequences of rLys and Lys were designed using the Reverse Translate Tool^3^ based on the preferential codon usage of *P. pastoris*^4^ (Supplementary Figure 1).

### Construction of Recombinant Plasmid pPICZαA-rLys/Lys and Transformation to *P. pastoris* X-33

Every DNA sequence composed of an *Xho*I restriction site, a *P. pastoris* Kex2 protease cleavage site, the rLys or Lys gene, two stop codons, and an *Xba*I restriction site. These specific genes were cloned into the plasmid of PUC19 in *Escherichia* coli DH5α. The rLys/Lys primers (rLys-F, 5′-CCGCTCGAGAAGAGAGCTGCTACTCATGAA-3′; rLys-R, 5′-GATTATTACTTAATAGTATCTCGACCCCAC-3′) and the genes were synthesized by Sangon Biotech (Shanghai, China). The plasmids containing the target genes were extracted and used as a PCR template for the amplification of the rLys gene. The amplified DNA fragments and plasmid pPICZαA were digested with *Xho*I and *Xba*I simultaneously. Connected the digested DNA and plasmid to construct a *Pichia* expression vector pPICZαA-rLys/Lys and transformed into *E. coli* DH5α. Positive sequences were identified and confirmed by PCR and DNA sequencing (Zhang et al., 2011).

The linearized pPICZαA-rLys vectors were transformed into *P. pastoris* X-33 by electroporation. The positive transformants were selected on YPDS plates with 100 μg/mL of Zeocin (Zhang et al., 2015).

### Expression of rLys/Lys in the 48-Well Plates, Shaking Flask, and Fermenter Level

In each mutant, 45 positive recombinants were selected and inoculated on 48-well plates in BMGY medium and cultured at 29°C, 250 rpm for 24 h, then methanol (100%) was added every 24 h to a final concentration at 0.5% (v/v). The supernatant was collected. Inhibition zone assay was used to select the recombinants with the highest activity. Expression at 1L shake flasks: The positive transformants were inoculated in 10 ml of YPD medium, containing 100 μg/mL of Zeocin and shaken until an optical density (OD)600 nm of 4.0–6.0 at 29°C and 250 rpm was obtained. The cells were harvested by centrifugation at 4000 rpm and 4°C for 5 min and resuspended to an OD600 nm of 1.0 in BMGY medium to induce the expression of the Lys/rLys at 29°C and 250 rpm. Methanol (100%) was added to a final concentration of 0.5%. The samples were centrifuged at 10,000 × *g* for a 120-h induction period, and every 24 h during that period, cells were removed from the centrifuge for 10 min and supernatant was subjected to Tricine-sodium dodecyl sulfate (SDS)–PAGE (Zhang et al., 2015).

Expression at 5 L bioreactors: A single colony from a positive transformant was incubated in 10 mL of YPD medium containing 100 μg/mL of Zeocin overnight at 29°C. A 2 ml cell suspension was inoculated into 200 mL YPD medium and shaken at 29°C for 16 h. Once the seed culture reached an OD600nm of 6.0 (late logarithmic growth phase), it was transferred into a 5-liter fermentor (BIOSTATB plus, Sartorius Stedim Biotech) and incubated as previously described. During the induction, the dissolved oxygen, temperature and pH were maintained at 40%, 29°C and 5.5, respectively. The total secreted protein levels in the fermentation broth were determined by the Bradford protein assay (Tiangen, Beijing, China) (Zhang et al., 2015).

### Purification of rLys/Lys

The fermentation supernatant was collected by centrifugation (5000 rmp, 30 min), filtered with micro-filtration membranes (0.45 μm), and dialyzed with 10 KDa cutoff dialysis tubing. The product was applied to a SP F.F. column. The protein samples were eluted by increasing the NaCl concentration in a stepwise manner. Firstly, the 100 mM NaCl, pH 7.5 was used to elute, then the concentration of NaCl was increased to 290 mM, and the eluent of corresponding elution peak was collected, dialyzed and freeze-dried (Zhang et al., 2015).

### Deglycosylation Experimental

The purified protein 127A, 127A232Q and Lys (100 μg) was dissolved into 40 μL H_2_O. A 5 μL of Deglycosylation Mix Buffer 2 was added into the protein liquid, and incubated at 75°C for 10 min, and then added 5 μL Protein Deglycosylation Mix II after cooling down, and mixed gently. The mixture was incubated at 25°C for 30 min, and then at 37°C for 1 h. Finally, SDS–PAGE was used to verify deglycosylation.

### Minimal Inhibitory Concentration (MIC) Assay

The MIC values of purified rLys/Lys were determined by the microtiter broth dilution method. Ten *Staphylococcus* standard strains and 5 clinical strains were used in this study. A clone of the strains was grown in 10 mL of Mueller-Hinton Broth (MHB) medium at 37°C, 250 rpm overnight. The overnight culture was transferred into a MHB medium to the final concentration at 1% (v/v), shaken at 37°C, 250 rpm, until OD600 nm reached 0.5, and diluted to 1 × 10^5^ CFU/mL with fresh MHB medium. Then purified rLys/Lys were two-fold serial dilutions from 1280 to 0.625 μg/mL with gradient concentration. A 90 μL of the bacteria suspension and 10 μL of serial concentration rLys/Lys were added in every well of 96-well plates. The 96-well plates were incubated at 37°C for 18–24 h. The MIC was defined as the lowest concentration of ones at which there was 99.9% bacteria were killed. All assays were performed in triplicate (Cao et al., 2015).

## Time-Kill Assay

*Staphylococcus aureus* ATCC 43300 strains were grown in MHB medium overnight at 37°C, 250 rpm. Fresh MHB medium was inoculated with 1% (v/v) of overnight culture and grown to mid-log phase (OD_600_nm = 0.5). The bacterial culture was diluted to 1 × 10^5^ CFU/mL with fresh MHB medium. The purified rLys/Lys was added in the bacterial culture diluted and the final concentrations were to 1×, 2×, or 4× MIC, respectively. The 100 μL of mixture was added in 12 wells of 24-well plates and cultured at 37°C, 250 rpm. The sample was taken out from each well at 0, 0.5, 1, 1.5, 2.5, 3.5, 5, 6.5, 8, 10, 12, and 24 h to determine the *in vitro* pharmacodynamics. Ampicillin was also tested with the same concentration gradient as control, and the fresh MHB was the negative control. All experiments were performed in triplicate, and the statistical analyses of the experimental data were done with Graphpad 7.0 (Yang et al., 2019).

### Synergism Assays of 127A, 127A232Q, Lys With Traditional Antibiotics

The chequerboard method was used for the measurement of interaction between the rLys/Lys and four kinds of antimicrobial agents with different mechanism including ampicillin, kanamycin, nisin, and ciprofloxacin. *S. aureus* ATCC 43300 was used in this assay. The rLys/Lys and antibiotics in a twofold dilution series were added into each well of the 96-well cell culture plates in a checkerboard fashion. The final concentrations of both rLys/Lys and antibiotics ranged from 1/16 to 8 × MIC to give a total volume in each well of 90 μL. Other conditions were the same as in MIC assay. The fractional inhibitory concentration index (FICI) was used to evaluate the effect of synergism of each combination. FIC of rLys/Lys = MIC of rLys/Lys in combination with antibiotic/MIC of rLys/Lys alone; FIC of antibiotics = MIC of antibiotic in combination with (rLys/Lys)/MIC of antibiotic alone; FICI = FIC of rLys/Lys+antibiotic. The result of interaction between two antimicrobial drugs was determined based on FICI: FICI ≤ 0.5 refers to synergy, 0.5 < FICI ≤ 1 refers to additivity, 1 < FICI ≤ 4 refers to indifference and FICI > 4 refers to antagonism. All experiments were performed in triplicate (Zheng et al., 2017).

### Cytotoxicity Assay and Selectivity Index

The RAW264.7 macrophage cells were used in the colorimetric MTT assay to test the cytotoxicity of rLys/Lys. Cells were resuspended to the final concentration at 2.5 × 10^5^ cell/mL and a 100 μL cell suspension was added into 96-well plates at 37°C for 24 h. A series of rLys/Lys solutions (100 μL) were added and incubated for another 24 h, 5 mg/L MTT solution (20 μL) was added into plates, incubated for 4 h in darkness. 150 μL Dimethyl sulfoxide (DMSO) was added into plates and the absorbance was measured at 570 nm with a spectrophotometer. Untreated cells were used as controls. The rate of inhibition of cell proliferation was calculated using the following formula: Cell viability (%) = (Abs570 nm of control-Abs570 nm of treated sample)/Abs570 nm of control × 100% (Yang et al., 2019). Selectivity index (SI) = IC_50_/MIC. The IC_50_ is the cell half maximal inhibitory concentration, which was calculated by the Graphpad Prism 7.0 (Basso et al., 2010).

### Hemolysis

Hemolytic activity of rLys/Lys to fresh mouse erythrocytes was assayed according to the standard method. In brief, a 100 μL peptide solution with a final concentration of 0–512 μg/mL was mixed with a 100 μL of 8% erythrocyte solution (diluted in 0.9% NaCl) and incubated for 1 h at 37°C. Cells were centrifuged at 1500 rpm for 5 min, the supernatant was collected, and the absorbance was measured at 540 nm. PBS and 0.1% Triton X-100 were used as the blank (A0) and positive (A100) control, respectively. Hemolysis of peptides was calculated by the following equation: Hemolysis (%) = [(ALys - A0)/(A100 - A0)] × 100.

### Effect of pH and Temperature on the Activity of 127A, 127A232Q

The MIC value was used to determine the pH and temperature stability of rLys/Lys. Five values of pH gradient solutions (100 mM) including glycine–HCl buffer (pH 2.0), sodium acetate buffer (pH 4.0), sodium phosphate buffer (pH 6.0), Tris–HCl buffer (pH 8.0), and glycine–NaOH buffer (pH 10.0) were used to incubate with rLys/Lys, at 37°C for 3h, respectively. The thermal stability of rLys/Lys was determined after a 1 h incubation at 4, 25, 40, 50, 60, 80, and 100°C in deionized water, separately. Deionized water served as independent controls. Subsequently, the antimicrobial activity of rLys/Lys against *S. aureus* ATCC 43300 was tested by MIC assays (Cao et al., 2015; Yang et al., 2017).

### Efficacy of rLys/Lys *in vivo*

#### Identification of Virulence Genes in *S. aureus* CVCC 546

The single clone of *S. aureus* CVCC 546 was cultured in 10 mL of MHB overnight at 37°C. The genomic DNA was extracted using a TIANamp Bacteria DNA kit. The target genes were amplified with the virulence genes primers (Stürenburg, 2009; Qin et al., 2016; Waryah et al., 2016; Jahanshahi et al., 2018; Table 1). The product was analyzed by electrophoresis.

**TABLE 1:** The primers of *Staphylococcus aureus* virulence genes.

Primer	Sequences (5′ to 3′)	Number of bases	Gene length (bp)
*mecA*	GTAGAAATGACTGAACGTCCGATAA	25	310
	CCAATTCCACATTGTTTCGGTCTAA	25	
*pvl*	ATCATTAGGTAAAATGTCTGGACATGATCCA	31	433
	GCATCAAGTGTATTGGATAGCAAAAGC	27	
*hla*	CTGATTACTATCCAAGAAATTCGATTG	27	209
	CTTTCCAGCCTACTTTTTTATCAGT	25	
*clfA*	ATTGGCGTGGCTTCAGTGCT	20	292
	CGTTTCTTCCGTAGTTGCATTTG	23	
*nuc*	ATGACAGAATACTTATTAAGTGCTGGC	27	360
	TGTATCAACCAATAATAGTCTGAATGT	27	
*sea*	GGTTATCAATGTGCGGGTGG	20	102
	CGGCACTTTTTTCTCTTCGG	20	
*psm-mec*	GAAGATCTATCACAAGATGAAATA	24	210
	ATGGATTTCACTGGTGTTATTACA	24	
*cna*	AAAGCGTTGCCTAGTGGAGA	20	192
	AGTGCCTTCCCAAACCTTTT	20	
*fnbpA*	GCGGAGATCAAAGACAA	17	1279
	CCATCTATAGCTGTGTGG	18	
*tsst-1*	ACCCCTGTTCCCTTATCATC	21	326
	TTTTCAGTATTTGTAACGCC	20	

### The Sepsis Mouse Model

Female BALB/c mice (6 weeks old, 25 ± 2 g) were purchased from the Beijing Vital River Laboratory Animal Technology Co., Ltd. All animal experiments were approved by the Laboratory Animal Ethical Committee in the Feed Research Institute of CAAS (AEC-CAAS-20090609). Operations were in accordance with ARRIVE guidelines.

### Absolute Lethal Dose of *S. aureus* ATCC 546 to Mice

Mice were divided into four groups, each with five. Different concentrations (5 × 10^8^, 10^9^, 5 × 10^9^, 10^10^ CFU/mL) of *S. aureus* CVCC 546 were diluted with saline. Each mouse was intraperitoneally injected with 200 μL diluted bacteria liquid. The absolute lethal dose was evaluated in 48 h after infection.

### Survival Rate of Mice

The mice were divided into four groups (5 mice per group) and injected with 5, 10 mg/kg of 127A, 127A232Q, Lys, C-Lys and 5, 10, 20 mg/kg of ampicillin at 1 h post infection (5 × 10^9^ CFU/mL of *S. aureus* CVCC 546, 200 μL). The survival rate was recorded daily for 72 h and the optimal therapeutic dose was identified.

### Bacteria Counts in the Tissue and Histological Section Experiment

Mice (5 mice per group) were challenged with *S. aureus* CVCC 546 (5 × 10^9^ CFU/mL, 200 μL) by intraperitoneal injection and treated with the 127A, 127A232Q, Lys, C-Lys (10 mg/kg) and ampicillin (20 mg/kg). Heart, liver, spleen and kidney were removed from mice at 24 h following the administration of *S. aureus* CVCC 546, and divided into two parts. One part of the tissues was homogenized in sterile PBS to the terminal concentration of 1 mg/mL to evaluate bacterial translocation by colony counting. All experiments were performed in triplicate. The other part of tissues was washed with PBS and fixed in paraformaldehyde. After they were washed with PBS, dehydrated with a graded series of ethanol (75–95%) and infiltrated with xylene and embedded in paraffin wax. Sections were cut, stained with hematoxylin and eosin and examined under a light microscope. A scoring standard is established to assess the degree of sample damage (Beijing Blue Sea Biological Technology Co., Ltd.) (Table 2).

**TABLE 2:** Tissue section score.

	PBS	CK	127A	127A232Q	Lys	C-Lys	Amp
Kidney	0	3	0	1	1	1	1
Spleen	0	3	1	1	3	1	2

*0, no inflammation; 1, very mild symptoms; 2, mild symptoms; 3, very serious. PBS, Uninfected group; CK, Untreated infection group.*

### Statistical Analysis

All data were presented as the means ± standard deviation (SD) and all statistical analyses were performed using GraphPad Prism software v8.0 (GraphPad Software, United States). Statistical significance of groups was analyzed using the one-way ANOVA and Tukey multiple comparison.

## Results

### Design of Synthetic Lysostaphin Genes

Two consensus N-linked glycosylation sites located in the lysostaphin sequence, one is “N125-S126-T127” and the other is “N232-K233-T234” (Figure 1). It was confirmed that the residue N125 was thought to be located at the C-terminal end of the catalytic domain, which was essential for the activity of lysostaphin (Zhao et al., 2014). The amino acid residue Q, with similar structure and properties, was used as substitution in this position. Similarly, Q was also used in position 232. To minimize the structural changes caused by mutations, P with the structure constrain effect and A with the small and neutral side chain were used in position 126 and 127. As results, seven Lys derived sequences (rLys) were designed to avoid the glycosylation in *P. pastoris* expression system (Supplementary Figure 1).

### Construction of Recombinant Plasmid pPICZαA-rLys/Lys

**FIGURE 2:**
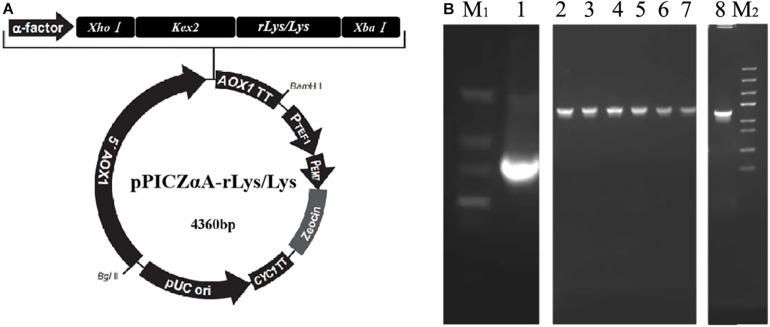
Construction of the pPICZαA-rLys/Lys plasmid. **(A)** The schematic diagram of the pPICZαA-rLys/Lys expression vector. **(B)** PCR products of the rLys/Lys. Line M1 and M2, DNA lander Marker II and Trans 5K Marker; line 1-8, the PCR products of 126P, 126P 232Q, 127A, 127A232Q, 125Q, 232Q, 125232Q.

The rLys sequences were digested with the restrict enzyme *Xho*I and *Xba*I, cloned into the pPICZαA vector digested with the same enzyme (Figure 2A), and transformed into *E. coli* DH5α. Positive recombinants were corrected by PCR and sequencing (Figure 2B). The recombinant plasmid pPICZαA-rLys/Lys was linearized with *Pme*I and transferred into *P. patoris* X-33 competent cells. The positive clones were survived on the YPDS plates with 100 μg/mL of Zeocin.

### Expression of rLys/Lys in the Shaking Flask Level

**FIGURE 3:**
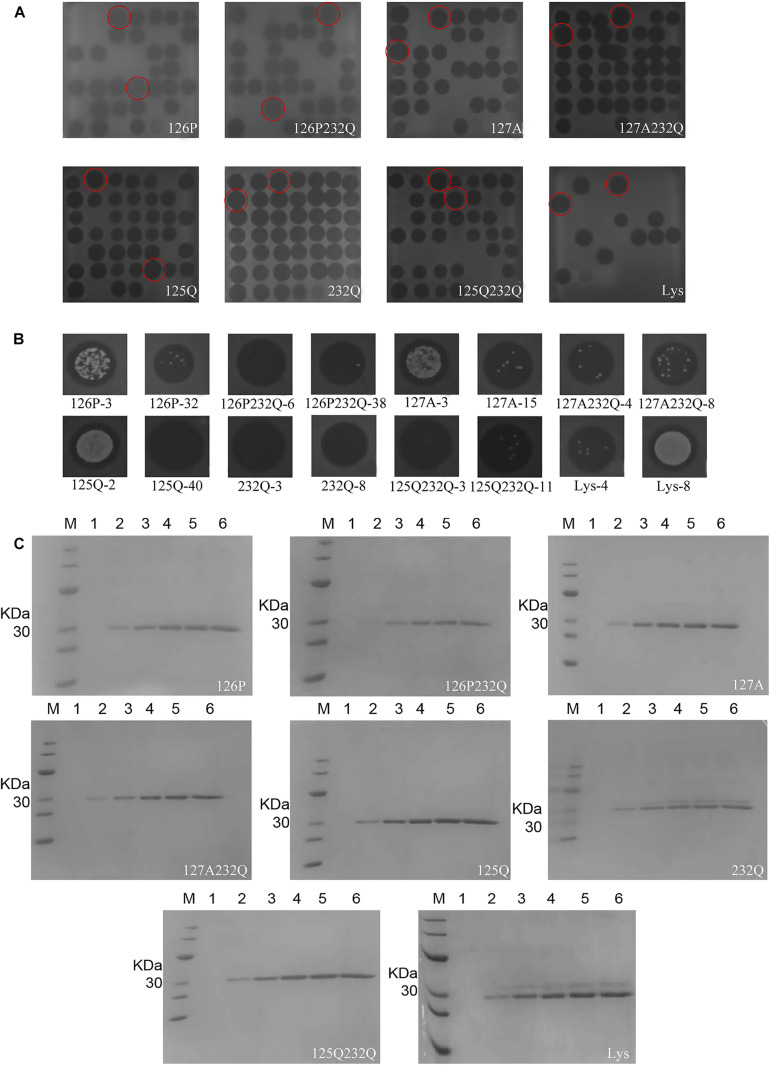
Expression of rLys/Lys in *P. pastoris* X-33 at the shaking flask level. The inhibition zones of rLys/Lys fermentation supernatants in **(A)** 48-well plates and **(B)** 1-L shake flasks. The superior transformants was emphasized by the red circles. **(C)** SDS–PAGE analysis of rLys/Lys fermentation supernatants in 1-L shake flasks. M, The molecular mass standards of Ruler I; line 1∼6, fermentation supernatants of rLys/Lys (10 μL) taken at 0, 24, 48, 72, 96, and 120 h of induction, respectively.

The transformants of 126P-3, 126P-32, 126P232Q-6, 126P232Q-38, 127A-3, 127A-15, 127A232Q-4, 127A232Q-8, 125Q-2, 125Q-40, 232Q-3, 232Q-8, 125232Q-3, 125232Q-11, Lys-4, Lys-8 were selected in 48-well plates by inhibition zone assay (Figure 3A). After expression in 1 L shaking flask, 126P-3, 126P232Q-6, 127A-15, 127A232Q-8, 125Q-40, 232Q-3, 125232Q-3, Lys-4 was selected for the further research (Figure 3B). As shown in Figure 3C, the target band was observed at 24 h induction, and the yield increased with the induction time. There were two bands in the nature Lys gene product in Figure 3C, one was the Lys and the other was the glycosylation product. The variants except 232Q had single band, indicating the glycosylation was successfully avoided in *P. pastoris.* The molecular weight of selected rLys/Lys were evaluated by MALDI-TOP MS. The weight of 126P (26989.013 Da), 126P232Q (26979.331 Da), 127A (26932.771 Da), 127A232Q (26936.814 Da), 125Q (26959.966 Da), 125 232Q (27032.595 Da) were consistent with the theoretical weight (27 KDa). However, there were two absorption peaks can be seen in the result of nature Lys. One peak is at 26956.751 Da, another is at 29438.351 Da, which was consistent with the molecular weight of the product of N-linked glycosylation. Meanwhile, two peaks were also observed in the result of 232Q. One of the peaks is at 26976.105 Da, another is at 29396.233 Da (Supplementary Figure 2), indicating that the site of 232 was not essential for the glycosylation in Lys.

### Antimicrobial Activity Assays to Bacteria

The rLys, Lys and commercial Lys (C-Lys) had potent antimicrobial activity to *Staphylococcus* sp. (Table 3). The MICs of the C-Lys to *S. aureus* were 0.07–4.74 μM. It was found that mutants 127A and 127A232Q had the increased activity with the MIC of 0.07–0.3 μM to *S. aureus*, and the antibiotic ampicillin was inferior to rLys and Lys. Additionally, the C-Lys showed low activity (MIC > 4.74 μM) to *S. epidermidi* CICC 10294, and the 127A and 127A232Q had very high activity with the MIC of 0.15 μM. However, compared with parental Lys, the mutants of 126P, 126P232Q, 125Q, 125Q232Q had lower activity to *S. aureus* CICC 10436, *S. epidermidis* CICC 10294 and *S. sciuri* 26. Due to the excellent activity of mutants 127A and 127A232Q, and there was no glycosylation in the preparation stage, they were selected for the further study.

**TABLE 3:** The MIC values of rLys/Lys.

Strain	MIC (μM)									
	
	126P	126P232Q	127A	127A232Q	125Q	125232Q	232Q	Lys	C-Lys	Amp
*S. aureus* ATCC 25923	0.30	0.30	0.15	0.30	0.30	0.15	0.15	0.15	0.30	< 0.31
*S. aureus* ATCC 43300	0.15	0.07	0.15	0.15	0.30	0.30	0.15	0.15	0.07	4.96
*S. aureus* CICC 10436	1.19	1.19	0.30	0.30	4.74	4.74	0.15	0.59	4.74	1.24
*S. aureus* CICC 10473	0.15	0.30	0.15	0.15	0.30	0.15	0.15	0.15	0.15	1.24
*S. aureus* CICC 21601	0.15	0.15	0.15	0.15	0.15	0.15	0.30	0.15	0.15	< 0.31
*S. aureus* CVCC 546	0.15	0.30	0.15	0.15	0.30	0.15	0.15	0.15	0.15	< 0.31
*S. aureus* FRI-GEL160701	0.15	0.30	0.15	0.15	0.30	0.30	0.15	0.15	0.15	< 0.31
*S. aureus* FRI-GEL180901	0.15	0.30	0.07	0.15	0.15	0.15	0.07	0.15	0.59	2.48
*S. epidermidis* CGMCC 1.4206	0.30	0.15	0.15	0.15	> 4.74	4.74	0.15	0.15	0.30	2.48
*S. epidermidis* CICC 10294	4.74	4.74	0.30	0.30	> 4.74	0.59	0.30	2.37	> 4.74	4.96
*S. hyicus* NCTC 10350	0.15	0.15	0.07	0.07	0.15	0.15	0.07	0.15	0.15	79.40
*S. hyicus* ACCC 61734	0.07	0.15	0.07	0.07	0.15	0.15	0.07	0.07	0.07	9.93
*S. sciuri* FRI-GEL180902	0.59	0.59	0.30	0.59	0.59	0.59	0.59	0.30	0.30	< 0.31
*S. sciuri* FRI-GEL180903	0.15	0.30	0.15	0.15	0.15	0.15	0.15	0.15	2.37	2.48

### Expression of 127A and 127A232Q in Fermentor Level

**FIGURE 4:**
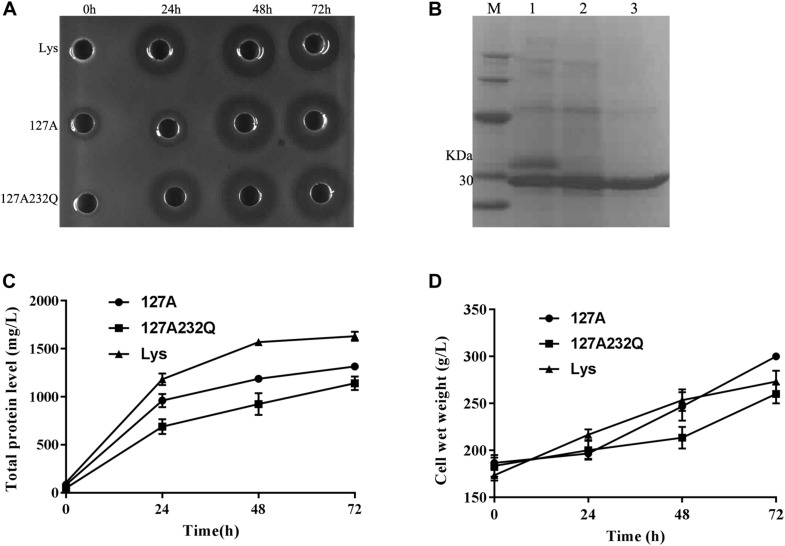
High-density cultivation of rLys/Lys in the fermentor level. **(A)** The inhibition zones of 127A, 127A232Q and Lys fermentation supernatants with different induced time against *S. aureus* ATCC 43300; **(B)** SDS–PAGE analysis of Lys, 127A and 127A232Q fermentation supernatants at 72 h. M, The molecular mass standards of Ruler I; line 1∼3, The fermentation supernatant of Lys, 127A and 127A232Q; **(C)** Time curves of the total secreted protein levels in the high-density fermentation; **(D)** Time curves of the cell wet weights in the high-density fermentation.

Transformants of 127A, 127A232Q and Lys were cultured and induced in 5-L fermentor at 29°C via high-density cultivation, respectively. The activity was detected after 24 h of induction and the total protein level increased with the inducing time. It is found that the mutants had the best activity during 48–72 h of induction (Figure 4A). After 72 h of fermentation, the total protein content of 127A, 127A232Q, and Lys were 1315, 1141, and 1631 mg/L, respectively (Figures 4B,C), and the cell wet weight increased to with the extension of the induction time (Figure 4D).

### rLys/Lys Purification

**FIGURE 5:**
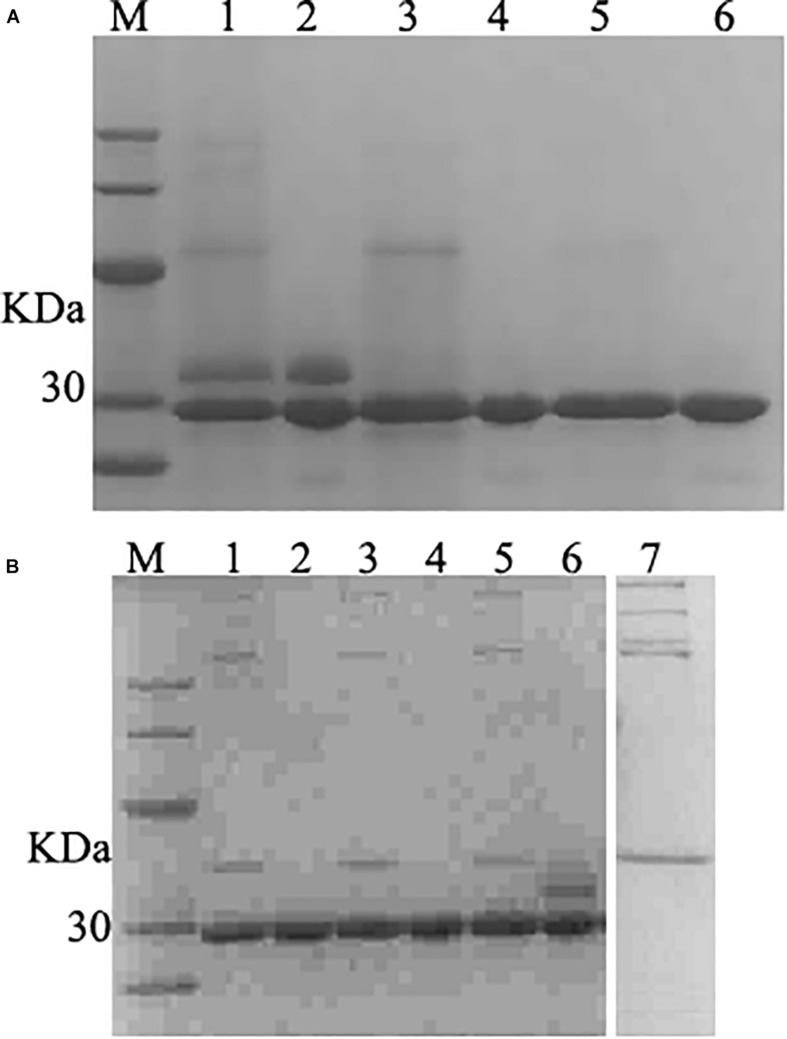
SDS–PAGE analysis of purified and deglycosylated rLys/Lys. **(A)** SDS–PAGE analysis of purified rLys/Lys. M, The molecular mass standards of Ruler I. Line 1, 3, 5, fermentation supernatants of Lys, 127A and 127A232Q; line 2, 4, 6, the purified target peak of Lys, 127A and 127A232Q (10 μL), respectively. **(B)** SDS–PAGE analysis of deglycosylation experiment. M, The molecular mass standards of Ruler I. Line 1,3,5, the deglycosylation of 127A, 127A232Q, Lys; Line 2,4,6, 127A, 127A232Q, Lys negative control (with no enzyme of deglycosylated protease); line 7, the control of deglycosylated protease.

The fermentation broth was centrifuged at 5000 rpm for 30 min and the supernatant was collected and filtered with 0.45 μm filtration. The membrane-packed dialysis was used to remove the salt to the conductivity of the supernatant was below 2 us/cm, and target proteins were purified with a cation-exchange column. The SDS-PAGE confirmed that purified product with the single band (Figure 5A). The product was freeze-dried a for further studied.

### Deglycosylation Experimental

127A, 127A232Q and Lys were subjected to deglycosylation experiment to confirm whether the variants were non-glycosylated and the additional upper band in SDS-PAGE of Lys was glycosylated (Figure 5A) from side chain of glycosylation. As shown in Figure 5B, the only band of 127A and 127A232Q has not changed, and showing the same weight as 127A control. The upper band corresponding for the glycolysated Lys disappeared, only the lower band was left. In general, the result indicated that the upper bands of Lys are glycosylated, while rLys is non-glycosylated.

### Time-Kill Assay of rLys/Lys

**FIGURE 6:**
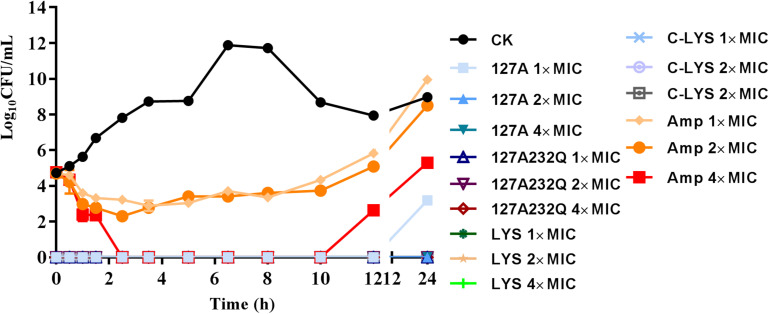
The time-killing curves of 127A, 127A 232Q, Lys and C-Lys against *S. aureus* ATCC 43300 *in vitro*. Ampicillin was used as the positive, CK: *S. aureus* ATCC 43300 were incubated in the presence of medium alone.

The time-killing kinetics showed that rLys/Lys exhibited effective antimicrobial activity against *S. aureus* ATCC 43300 *in vitro* (Figure 6). The rLys/Lys had significant advantages in antimicrobial activity and sterilization speed compared with ampicillin. For 1 × MIC, 2 × MIC, 4 × MIC of 127A, 127A232Q, Lys, and C-Lys treatment groups, the bacteria were almost completely killed within 30 minutes. There was no regrowth except the 1 × MIC of 127A in 24 h. However, for ampicillin treatment group, the Log_10_ (CFU/mL) of *S. aureus* decreased to 2.5 to 3.5 within 1.5 h. While the bacteria slowly recovered and regrew in 2 to 10 h in 1 and 2 × MIC of ampicillin.

### Synergism Assays

The drug combination assay was applied to detect the synergism against pathogenic bacteria (Table 4). 127A, 127A232Q, Lys and C-Lys were observed in combination with four antibacterial drugs (ampicillin, kanamycin, nisin, and ciprofloxacin) with different mechanisms against *S. aureus* ATCC 43300. The combination of 127A with the four antibiotics showed FICI values of 1.125, 1, 1, 1.0625, respectively; and the FICI indexes between 127A232Q and the four antibiotics were 1.0625, 1, 1, 1.0625, respectively. According to the synergy index, it was found that all lysostaphins or non-glycosylation engineered enzymes had additive and indifference effects with the four antibiotics.

**TABLE 4:** Combination effects of 127A, 127A232Q, Lys, C-Lys with antibiotics.

Combination	Variety	*S. aureus* ATCC 43300			
		
		MIC_a_	MIC_c_	FIC	FICI
127A-Amp	127A	0.07	0.01	0.125	1.125
	Amp	2.48	2.48	1	
127A-Kan	127A	0.07	0.04	0.5	1
	Kan	132.23	66.12	0.5	
127A-Nisin	127A	0.07	0.04	0.5	1
	Nisin	36.57	18.29	0.5	
127A-Cip	127A	0.07	0.004	0.0625	1.0625
	Cip	0.76	0.76	1	
127A 232Q-Amp	127A 232Q	0.15	0.01	0.0625	1.0625
	Amp	2.48	2.48	1	
127A 232Q-Kan	127A 232Q	0.15	0.07	0.5	1
	Kan	132.23	66.12	0.5	
127A 232Q-Nisin	127A 232Q	0.15	0.07	0.5	1
	Nisin	36.57	18.29	0.5	
127A 232Q-Cip	127A 232Q	0.15	0.01	0.0625	1.0625
	Cip	0.76	0.76	1	
Lys-Amp	Lys	0.15	0.07	0.5	1
	Amp	2.48	1.24	0.5	
Lys-Kan	Lys	0.15	0.07	0.5	1
	Kan	132.23	66.12	0.5	
Lys-Nisin	Lys	0.15	0.07	0.5	1
	Nisin	36.57	18.29	0.5	
Lys-Cip	Lys	0.15	0.01	0.0625	1.0625
	Cip	0.76	0.76	1	
C-Lys-Amp	C-Lys	0.15	0.02	0.125	1.125
	Amp	2.48	2.48	1	
C-Lys-Kan	C-Lys	0.15	0.07	0.5	1
	Kan	132.23	66.12	0.5	
C-Lys-Nisin	C-Lys	0.15	0.07	0.5	1
	Nisin	36.57	18.29	0.5	
C-Lys-Cip	C-Lys	0.15	0.01	0.0625	1.0625
	Cip	0.76	0.76	1	

*MIC_*a*_, the MIC of variety drug alone (including Lys, rLys and antibiotic alone); MIC_*c*_, the MIC of the most effective combination. Amp, Ampicillin; Kan, Kanamycin; Ciprofloxacin.*

### Cytotoxicity and Selectivity Index

**FIGURE 7:**
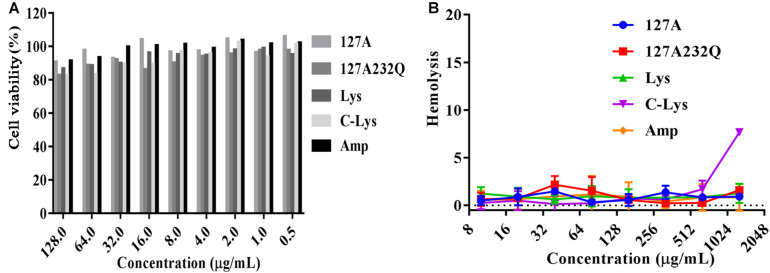
The cytotoxicity and hemolysis of rLys/Lys. **(A)** Cytotoxicity of 127A, 127A232Q, Lys, C-Lys and Ampicillin against RAW264.7 cells; **(B)** Hemolytic activity of 127A, 127A232Q, Lys, C-Lys and Ampicillin against fresh mouse red blood cells.

The cytotoxicity of rLys, Lys, C-Lys was evaluated by measuring the cell viability of lysostaphins treated mouse RAW264.7 cells. As shown in Figure 7A, with the increasing concentration of lysostaphin, the cell viability decreased slightly. When exposed to 128 μg/mL of 127A, 127A232Q, Lys, and C-Lys, the cell viability were 91.1, 83.1, 87.1, and 82.8%, respectively. And the viability of cells was higher than 90% at lower concentrations of the lysostaphins with the concentration of 1–64 μg/mL. The results suggested that lysostaphins had low cytotoxicity against RAW264.7 cells. To determine the safe range of drug effect, the SI values should be higher than 10. The higher selectivity index of 127A and 127A232Q (SI = 101.2 and 1567.3) (Supplementary Table 1) encouraged us study further.

### Hemolysis

The hemolytic assay mainly detects whether the peptide is toxic to the red blood cell. As shown in Figure 7B, only C-Lys has the highest concentration at 1280 μg/mL. Red blood cells have no organelles, and the main barrier is the cell membrane. The hemolytic experiment fully verified that the lysostaphins did not damage the cell membrane, having the security in blood.

### Effect of pH and Temperature on the Activity of rLys and Lys

The pH and thermal stability of rLys/Lys were shown in Table 5. rLys/Lys displayed strong stability in different pH values from 2.0 to 8.0 against *S. aureus*, and the activity of 127A and 127A232Q lightly reduced in the alkaline environment (pH 10.0). Meanwhile, it was found that the temperature has a great influence on lysostaphin. The activity of rLys, Lys, C-Lys remained unchanged in the temperature range from 20–60°C, after exposure to 80°C and 100°C, the MIC values of 127A increased 3.9 and 31.6 folds, 127A232Q increased 7.9 and 31.6 folds.

**TABLE 5:** The pH and temperature stability.

	MIC (μM)											
	
	pH					Temperature (°C)						
		
	2.0	4.0	6.0	8.0	10.0	4	25	40	50	60	80	100
127A	0.15	0.15	0.15	0.15	0.30	0.07	0.15	0.15	0.15	0.15	0.59	4.74
127A232Q	0.15	0.15	0.15	0.15	0.30	0.15	0.15	0.15	0.15	0.15	1.19	4.74
Lys	0.15	0.15	0.15	0.15	0.15	0.15	0.15	0.15	0.15	0.15	0.30	0.59
C-Lys	0.15	0.15	0.15	0.15	0.15	0.15	0.15	0.15	0.15	0.15	> 4.74	>4.74
Amp	9.93	9.93	9.93	9.93	9.93	9.93	9.93	9.93	9.93	9.93	9.93	9.93

### Efficacy of rLys/Lys *in vivo*

#### Absolute Lethal Dose of *S. aureus* ATCC 546 to Mice

**FIGURE 8:**
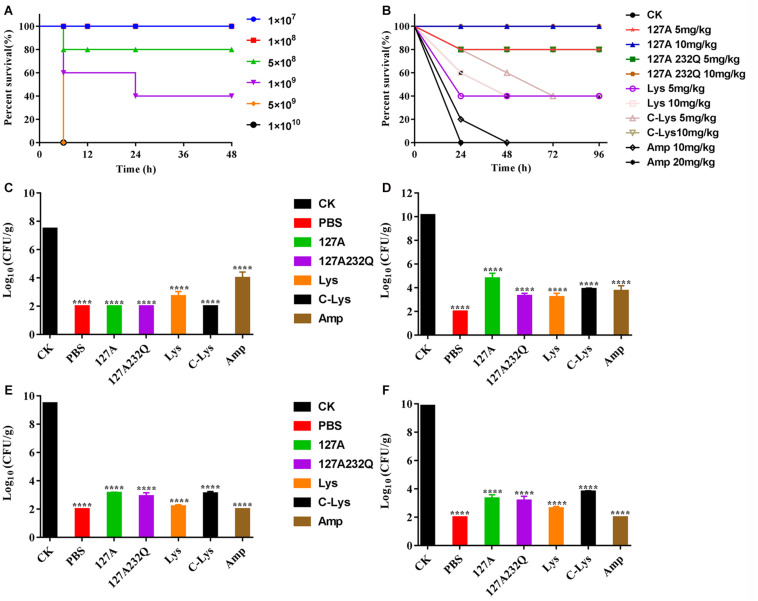
Absolute lethal dose of *Staphylococcus aureus* CVCC 546 and protection efficacy of rLys/Lys in mice. **(A)** The absolute lethal dose of *S. aureus* CVCC 546 to mice. **(B)** Survival of mice treated with rLys, Lys, C-Lys in *S. aureus* lethal models. CK: *S. aureus* injected, untreated. **(C–F)** Effect of 127A, 127A232Q, Lys, C-Lys and Amp on bacterial burdens of blood, liver, kidney and spleen in *S. aureus*-infected mice. CK, Untreated infection group; PBS, Uninfected group; Amp, Ampicillin. All data were analyzed by the one-way ANOVA and Bonferroni multiple comparison. ****p < 0.0001. The results are given as the mean ± SD (*n* = 3).

After intraperitoneal injection *S. aureus* ATCC 546 (Figure 8A), the mice of low-dose injection groups (1 × 10^7^, 1 × 10^8^ CFU/mL) were alive within 72 h. The mice of high-dose injection groups showed typical symptoms of infection: eyes secreted mucus, smaller eyelid opening, the body temperature increased, and severe shivered and shaked. The survival rate of mice injected with 5 × 10^8^, 1 × 10^9^ CFU/mL *S. aureus* were 40% and 80%, respectively. When the challenge dose of *S. aureus* was 5 × 10^9^, 1 × 10^10^ CFU/mL, all mice died within 6 hours. Therefore, 5 × 10^9^ CFU/mL of *S. aureus* was determined as the absolute lethal dose.

### The Therapeutic Effect of rLys, Lys, C-Lys

After intraperitoneal injection of the absolute lethal dose of *S. aureus* ATCC 546, the mice were treated with different concentrations of lysostaphin by intraperitoneal injection. The survival rates of the mice were shown in Figure 8B. All of the mice in untreated group died within 24 h. Although 10 mg/kg Ampicillin failed to improve the survival rate, it prolonged the survival time of the mice. And the 5 mg/kg Lys, 10 mg/kg Lys, 5 mg/kg C-Lys, and 20 mg/kg Ampicillin can make the survival rate of mice reach 40%. The mice survival rates of 5 mg/kg 127A, 5 mg/kg 127A232Q and 10 mg/kg C-Lys treatment groups were up to 80%, and they had the similar therapeutic effects. 127A and 127A232Q at a concentration of 10 mg/kg had the best effect which can make all mice survive. It’s demonstrated that lysostaphin showed a good therapeutic effect, which at the same time, its drug efficacy was more remarkable than that of antibiotic treatment group.

### The Effect of rLys, Lys, C-Lys on the Bacterial Load

The bacterial loads were counted in different organs. As shown in the Figures 8C–F, blood, liver, kidney and spleen were taken at 24 h post-treatment with 10 mg/kg rLys, Lys and C-Lys. The bacteria in blood were almost completely eliminated after treatment with 127A, 127A232Q and C-Lys, however, there are still had about 5 log CFU after treatment with Amp. The bacteria of each examined organ of liver, kidney and spleen showed 5-7 log CFU reduction, and the bacterial load had all been reduced by 99.99%. In general, all lysostaphins show highly bactericidal activity in various tissues.

### Histopathological Observation

**FIGURE 9:**
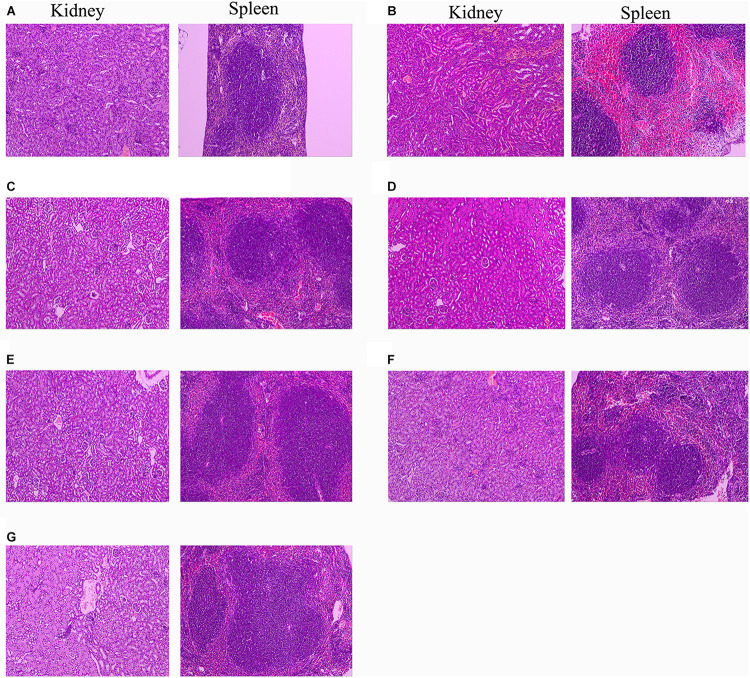
Protection of rLys/Lys against multiple-organ injuries from *S. aureus* CVCC 546. **(A)** Uninfected group; **(B)** Untreated infection group; **(C–G)** Therapeutic effect of 127A, 127A232Q, Lys, C-Lys and Amp (10 mg/kg) on kidney and spleen injury.

Since 10 mg/kg lysostaphin can provide a higher survival rate of mice, the HE staining analysis of each tissue of the mice treated with this dose drug was performed. The kidney and spleen of uninfected mice have no pathological symptoms (Figure 9A). In the untreated infection group (Figure 9B), the local renal tissue interstitium was infiltrated with scattered inflammatory cells. A large area of renal tubules was atrophy and degeneration with obvious local inflammation. A small amount of glomerular atrophy was observed at the obvious local inflammatory sites and the tissue section was scored as “very severe” (Table 2). The red pulp of the spleen tissue of the untreated infection mice was congested, the white pulp volume relatively increased, the splenic nodules were enlarged, the center of occurrence was obvious, the marginal zone was clearly visible, and obviously enlarged, the splenic cord atrophy, disappeared locally, and a large number of splenic sinuses red blood cells, splenic cord lymphocytes were depleted and the tissue section was scored as “very severe.”

After treatment with 127A, 127A232Q, C-Lys, Lys and ampicillin, the kidney tissue showed obvious recovery. The therapeutic effect of the drug was evaluated as: 127A > 127A232Q = C-Lys > Lys = Amp. In 127A treatment group, kidney tissue was recovered almost the same as normal within 24 h. And the Lys, which was the least effective group, also could relieve symptoms, with local inflammatory cell infiltration, renal tubular atrophy, and the disappearance of glomerular involvement. In the spleen tissue of the treatment group, the drug treatment effect was evaluated as: 127A = 127A232Q = C-Lys > Amp > Lys. Among them, 127A can make the symptom score reach the “mild symptom” level.

## Discussion

Glycosylation is the process of adding sugars to proteins or lipids under the control of enzymes which starting at the endoplasmic reticulum and ending at the Golgi apparatus (Zhang et al., 2019). There are 5 kinds of glycosylation, including N-glycosylation, O-glycosylation, and the rarely ones such as C-glycosylation, S-glycosylation and P-glycosylation (Eichler, 2019). Among them, N-glycosylation is the process that the sugar chains connect to the free -NH_2_ group of the specific asparagine (NXS/T, X indicates any amino acid except proline) in the nascent peptide chain (Marshall, 1974; Bause and Hettkamp, 1979; Han et al., 2020); O-glycosylation is the process that the carbon chains transfer to the oxygen atom of the hydroxyl group of serine, threonine or hydroxylysine in the polypeptide chain, and its glycosylation positions are highly selective (Stavenhagen et al., 2019; Wells and Feizi, 2019). In this study, there are two N-glycosylation sites at 125 (NST) and 232 (NKS). These two sites of amino acids were modified with similar amino acid residue Q. Meanwhile, because of the key role of 125N at the C-terminal end of the catalytic domain, the mutants of 126S and 127T were also designed. The result showed that the mutants of 125Q could remove the glycosylation effectively, while the mutant of 232Q could not (Figure 3). It was found that the N-glycosylation efficiency was decreased within 60 AA of the C-terminus (Nilsson and von Heijne, 2000). The distance from the 232N site to the C-terminus of lysostaphin is only 14 residues, which leading to the inefficient glycosylation in this position (Figure 3C). Additionally, the N-glycosylation frequency was high when the ± 1 site of the glycosylation was S. The ± 1 sites of 125N in lysostaphin were both S, and the ± 1 sites of 232N were K and W. The sequence of the N-glycosylation sites in lysostaphin suggesting that 125N is a favorable glycosylation site of recombinant lysostaphin in eukaryotic *P. pastoris* expression system (Hamby and Hirst, 2008).

Seven modified rLys (126P, 126P232Q, 127A, 127A232Q, 125Q, 232Q, 125232Q) were obtained by *P. patoris* expression. Compared with all of the MICs of rLys, the result showed that the MIC values of 125Q was slightly higher than the other rLys (1-4 times) against *S. aureus*, *S. epidermidis* and *S. hyicus*. Zhao et al. (2014) also concluded that lysozyme activity was reduced by replacing the N-amino acid at position 125 with Gln. Although no relevant reference has investigated the role of loop at 125 amino acids in the Lys structure (Sabala et al., 2014; Tossavainen et al., 2018), the results demonstrated that the conserved site at position 125 has an effect on the activity of lysostaphin. In addition, the modified of amino acids on position 126 and 127 (126P, 126P232Q, 127A, 127A232Q) can effectively inhibit the formation of glycosylation and the antimicrobial activity of them were 1–15.8 times improved compared to Lys and C-Lys.

The antibacterial activity of the rLys, Lys and C-Lys was superior to that of ampicillin. Low concentration (1 × MIC) of 127A, 127A 232Q, Lys, C-Lys can effectively kill *S. aureus* in few minutes. However, 2 × MIC ampicillin couldn’t completely inhibit bacteria, and after treatment with 4 × MIC ampicillin the bacteria rapidly regrew after 10 h. The rapid bactericidal ability of lysostaphin owed to the character of effective hydrolyze staphylococcal cell wall peptidoglycans. C-terminal cell-wall-targeting domain promotes lysostaphin binding to staphylococcal peptidoglycan (Baba and Schneewind, 1996) and the N-terminal domain with glycylglycine endopeptidase activity cleaves pentaglycine cross bridges (Schindler and Schuhardt, 1964). The activity of Lys expressed in *P. patoris* was equivalent or slightly stronger than that of C-Lys expressed in *E. coli*, this preponderant antimicrobial activity may owe to the *P. pastoris* eukaryotic expression system which could promote the proper folding and reduce protease hydrolysis of exogenous target protein (Yang and Zhang, 2018).

The synergistic activity of 127A, 127A232Q, Lys, C-Lys with various drugs was determined in this study. Due to the complementarity of mechanism, the lysostaphin did not show antagonistic action to various types of drugs. For instance, the ampicillin inhibited the activity of transpeptidase, making it impossible to transpeptidase and peptidoglycan cross-linking inhibiting the formation of cell wall. And ciprofloxacin is a small molecule antibiotic (331 Da) (Park and Strominger, 1957), which entered into bacteria and acted on DNA topoisomerase and inhibited DNA replication (LeBel, 1988). Furthermore, the 127A, 127A232Q, Lys and C-Lys exhibited additive effect with kanamycin and nisin. The bactericidal action of kanamycin and nisin is non-destructive cell wall mechanisms. The kanamycin is binding to 30S subunit of bacterial ribosome to inhibit bacterial protein synthesis (Becker and Cooper, 2013; Wargo and Edwards, 2014) and nisin is acting on bacterial membrane to form holes (Montville and Chen, 1998; Chu et al., 2010). Kanamycin and nisin have synergistic effect with bactericides that act on cell walls during bacterial reproduction; it provided evidence for the additive effect of lysozyme and Kanamycin (Chai et al., 2015). Therefore, it is speculated that the probable reason of additive effect is that lysostaphin firstly destroys cell wall, allowing antibiotics further interact with cell membrane or intramembrane macromolecules. Meanwhile, the exact mechanism of synergistic activity for lysostaphin with antibiotics *in vitro*/*vivo* should be further study.

Stability is an important index for the production and application of lysostaphin, previous study showed the optimum pH for the recombinant lysostaphin antimicrobial activity was at 7.0–9.0 (Sharma et al., 2006). In this study, lysostaphin has strong stability at different pH ranging from 2 to 8, and the antimicrobial activity was slightly reduced (MIC from 0.15 to 0.3 μM) in alkaline environment (pH 10.0). Moreover, the activity of lysostaphin showed stable within 60°C (1 h treatment). The temperature had less effect on the activity of 127A, 127A232Q and Lys (activity reduced 3.9, 7.9, and 2 times, respectively) than that of C-Lys after 1 h heat treatment at 80°C (activity reduced by 31.6 times). Sharma et al. (2006) indicated that recombinant lysostaphin expressed in *E. coli* showed low temperature stability (40% activity lost after 10 min heat treatment at 60°C). The difference of temperature stability may be related to the structural stability. The commercial lysostaphin exhibited two melting temperature (*Tm*: at 47°C and 60°C), and lysostaphin variants S126P and T127A showed a single apparent *Tm* (Zhao et al., 2014). In consequence, lysostaphin expressed in *P. pastoris* in this study also displayed higher stability than that of expressed in *E. coli* (C-Lys).

High virulent *S. aureus* CVCC 546 contains many virulence genes, including *pvl* (dissolve cell membranes), *nuc* (Solubilize DNA), *sea* (cause multiple organ damage), *psm-mec* (regulate biofilm) (Queck et al., 2009; Kaito et al., 2011) and *cna* (collagen binding protein) (Supplementary Figure 3), which is easy to cause organ damage and death of animals after infected. Meanwhile, *S. aureus* CVCC 546 showed multidrug resistant against tetracycline, bacitracin and sulfamethoxazole (Wang et al., 2018), this brings some limitations to antibiotic therapy. In this study, all mice were died after challenged with 5 × 10^9^ CFU/mL *S. aureus* CVCC 546 within 6 h (Figure 8). 127A, 127A232Q, Lys, and C-Lys administered once a day at 10 mg/kg can make the survival rate increased to 100, 100, 40, and 80%, respectively, and significantly clear the amount of *S. aureus* in spleen (99.99%) for 23 h (Figure 8). The 20 mg/kg ampicillin, administered twice a day at 20 mg/kg, showed only 40% survival rate, the therapeutic effect was far less than that of lysostaphin. By histological observation (Figure 9), 127A showed the best protective effect on organs, which was consistent with the therapeutic effect.

Previous study has proved that the *in vitro* short serum half-life period of lysostaphin (<1 h) (Walsh et al., 2003) did not affect the capacity of lysostaphin *in vivo* (Kokai-Kun et al., 2007). Lysostaphin not only protected mice from *S. aureus* infection (Kokai-Kun et al., 2007; Chen et al., 2014), but also treated rabbit and dog endocarditis caused by *S. aureus* (Goldberg et al., 1967). Meanwhile, lysostaphin can also be used as a topical drug for wound infection (Cheleuitte-Nieves et al., 2020). Additionally, studies demonstrated that β-lactam antibiotics oxacillin and lysostaphin combination could reduce lysostaphin dosage from 5 to 1 mg/kg for treatment of an MRSA infection (Kiri et al., 2002). In addition, lysostaphin in combination with nisin was effective in killing most biofilm of *S. aureus* involved in bovine mastitis (Ceotto Vigoder et al., 2016), the results were consistent with synergy in this study (Table 4). The combination of antibiotics and lysostaphin is superior to the use of a single drug, since it would prevent the possible development of lysostaphin-resistant (Bastos et al., 2015).

In general, the lysostaphin mutants were designed to eliminate the glycosylation during the expression in *P. pastoris.* In these mutants, 127A and 127A232Q showed the potent antimicrobial activity to *S. aureus*, and they killed MRSA strain ATCC 43300 in very short time (99.9% killing rate within 30 min and no regrowth in 24 h). The 127A and 127A232Q showed a very low toxicity and a high stability at the wide scope of pH and temperature. They could be mixed used with types of drugs to reduce the usage of traditional antibiotics. Additionally, 127A and 127A232Q showed stronger protective effect against *S. aureus* than natural Lys and commercial Lys in mice. These results indicated the non-glycosylated lysostaphin is a potential effective drug for clinical treatment of *S. aureus* infection.

## Data Availability Statement

The original contributions presented in the study are included in the article/Supplementary Material, further inquiries can be directed to the corresponding author/s.

## Author Contributions

RM and JW conceived and designed experiments. WS carried out all the experiments. DT, NY, and XM prepared partial materials in laboratory. WS, RM, and NY contributed in writing. JW contributed in funding acquisition. YH contributed to materials and reagents. All authors contributed to the article and approved the submitted version.

## Conflict of Interest

The authors declare that the research was conducted in the absence of any commercial or financial relationships that could be construed as a potential conflict of interest.

## Footnotes

1 http://amr-review.org

2 http://www.cbs.dtu.dk/services/NetNGlyc/

3 http://bioinformatics.org//sms2/rev_trans.html

4 http://www.kazusa.or.jp/codon/
