# Supplementary material for: Design and High Expression of Non-glycosylated Lysostaphins in Pichia pastoris and Their Pharmacodynamic Study
Source: Front Microbiol. 2021 Mar 18;12:637662. doi: 10.3389/fmicb.2021.637662 (PMC8012855; doi:10.3389/fmicb.2021.637662)
Supplement: Supplementary file 1 [file Data_Sheet_1.pdf]

Supplementary Material

Design and high expression of nonglycosylated lysostaphins in *Pichia pastoris* and their pharmacodynamic study

Wenluan Shen [1,2*], Na Yang [1,2*], Da Teng[1,2], Ya Hao [1,2], Xuanxuan Ma[1,2], Ruoyu Mao [1,2] and Jianhua Wang [1,2**]**

[1] Gene Engineering Lab, Feed Research Institute, Chinese Academy of Agricultural Science, Beijing 100081, P. R. China

[2] Key Laboratory of Feed Biotechnology, Ministry of Agriculture and Rural Affairs, Beijing 100081, P. R. China

*** Co-first author:**

Wenluan Shen and Na Yang contributed equally to this work.

**** Correspondence:**

Ruoyu Mao: maoruoyu@caas.cn

Jianhua Wang: wangjianhua@caas.cn; 2681298635@qq.com

```
  XhoI
CCGCTCGAGAAGAGAGCTGCTACTCATGAACATTCTGCTCAATGGTTGAACAACTACAAG
       E  K  R  A  A  T  H  E  H  S  A  Q  W  L  N  N  Y  K
          Kex2
AAGGGTTACGGTTACGGTCCATACCCATTGGGTATTAACGGTGGTATGCATTACGGTGTT
 K  G  Y  G  Y  G  P  Y  P  L  G  I  N  G  G  M  H  Y  G  V
GATTTTTTTATGAACATTGGTACTCCAGTTAAGGCTATTTCTTCTGGTAAGATTGTTGAA
 D  F  F  M  N  I  G  T  P  V  K  A  I  S  S  G  K  I  V  E
GCTGGTTGGTCTAACTACGGTGGTGGTAACCAAATTGGTTTGATTGAAAACGATGGTGTT
 A  G  W  S  N  Y  G  G  G  N  Q  I  G  L  I  E  N  D  G  V
CATAGACAATGGTACATGCATTTGTCTAAGTACAACGTTAAGGTTGGTGATTACGTTAAG
 H  R  Q  W  Y  M  H  L  S  K  Y  N  V  K  V  G  D  Y  V  K
GCTGGTCAAATTATTGGTTGGTCTGGTTCTACTGGTTACTCTACTGCTCCACATTTGCAT
 A  G  Q  I  I  G  W  S  G  S  T  G  Y  S  T  A  P  H  L  H
TTTCAAAGAATGGTTAACTCTTTTTCTAACTCTACTGCTCAAGATCCAATGCCATTTTTG
 F  Q  R  M  V  N  S  F  S  N  S  T  A  Q  D  P  M  P  F  L
AAGTCTGCTGGTTACGGTAAGGCTGGTGGTACTGTTACTCCAACTCCAAACACTGGTTGG
 K  S  A  G  Y  G  K  A  G  G  T  V  T  P  T  P  N  T  G  W
AAGACTAACAAGTACGGTACTTTGTACAAGTCTGAATCTGCTTCTTTTACTCCAAACACT
 K  T  N  K  Y  G  T  L  Y  K  S  E  S  A  S  F  T  P  N  T
GATATTATTACTAGAACTACTGGTCCATTTAGATCTATGCCACAATCTGGTGTTTTGAAG
 D  I  I  T  R  T  T  G  P  F  R  S  M  P  Q  S  G  V  L  K
GCTGGTCAAACTATTCATTACGATGAAGTTATGAAGCAAGATGGTCATGTTTGGGTTGGT
 A  G  Q  T  I  H  Y  D  E  V  M  K  Q  D  G  H  V  W  V  G
TACACTGGTAACTCTGGTCAAAGAATTTACTTGCCAGTTAGAACTTGGAACAAGTCTACT
 Y  T  G  N  S  G  Q  R  I  Y  L  P  V  R  T  W  N  K  S  T
AACACTTTGGGTGTTTTGTGGGGTACTATTAAGTAATAATCTAGAGC
 N  T  L  G  V  L  W  G  T  I  K  *  *
                                        XbaI
```

Supplementary Figure 1. The optimized gene sequences of lysostaphins. The restriction sites and Kex 2 cleaving site were marked. The yellow shade was signed for the glycosylation sites to modify. The termination codon was marked with asterisks.

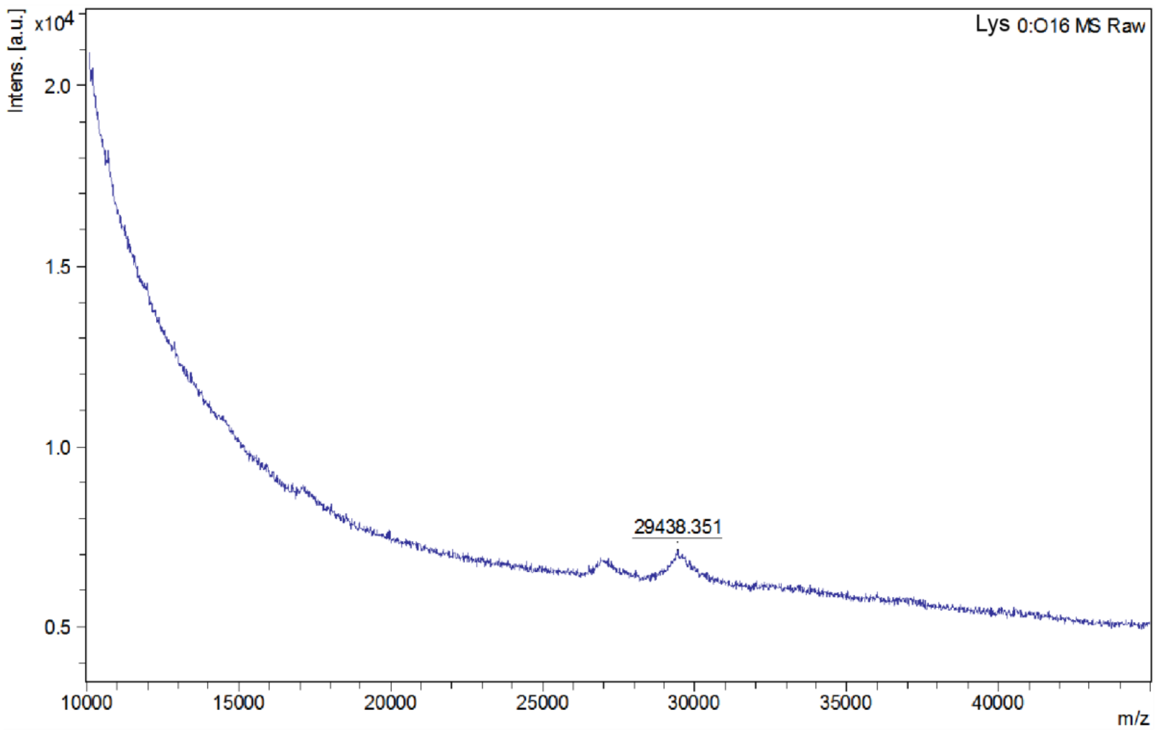

Lys 0:O16 MS Raw
Intens. [a.u.]
x10^4
2.0
1.5
1.0
0.5
29438.351
10000
15000
20000
25000
30000
35000
40000
m/z

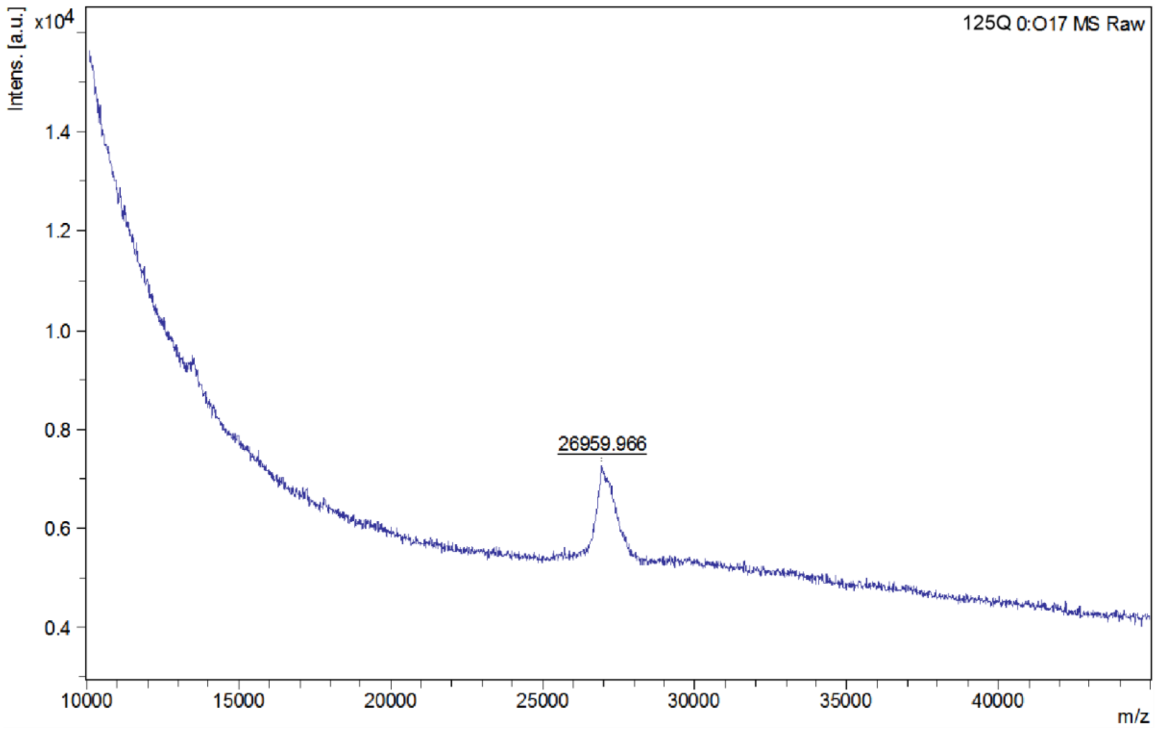

125Q 0:O17 MS Raw
Intens. [a.u.]
x10^4
1.4
1.2
1.0
0.8
0.6
0.4
26959.966
10000
15000
20000
25000
30000
35000
40000
m/z

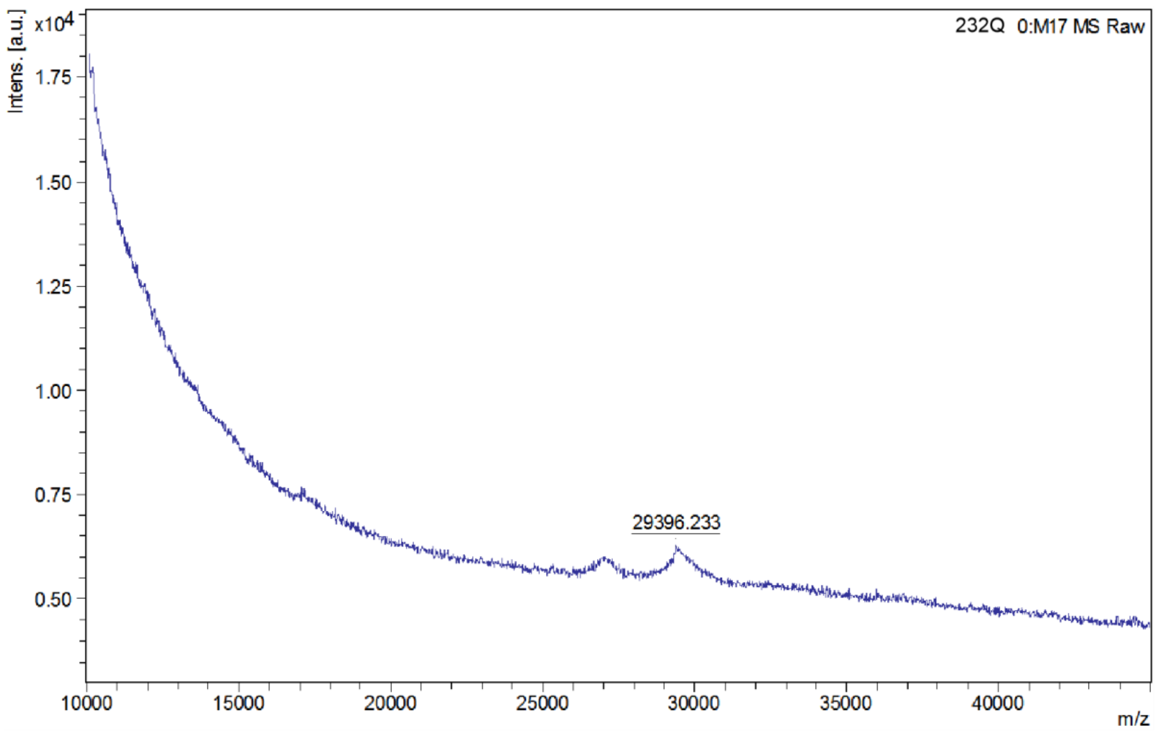
232Q 0:M17 MS Raw
x10^4
Intens. [a.u.]
1.75
1.50
1.25
1.00
0.75
0.50
29396.233
10000
15000
20000
25000
30000
35000
40000
m/z

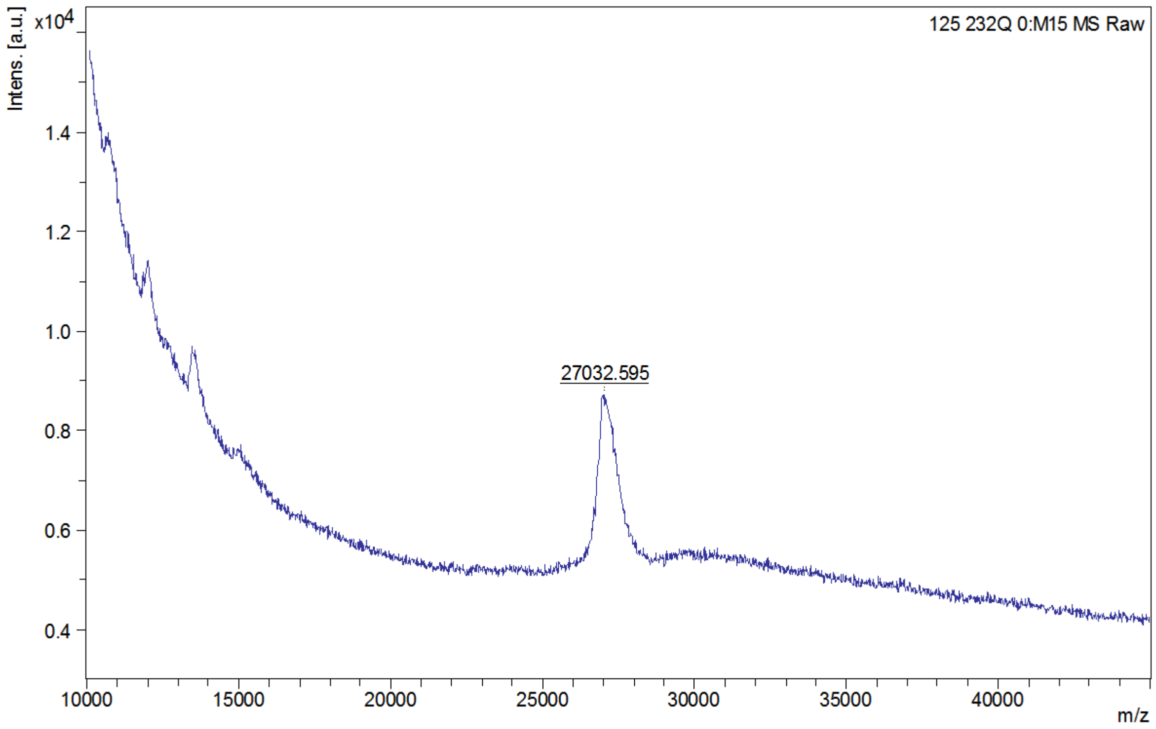
125 232Q 0:M15 MS Raw
x10^4
Intens. [a.u.]
1.4
1.2
1.0
0.8
0.6
0.4
27032.595
10000
15000
20000
25000
30000
35000
40000
m/z

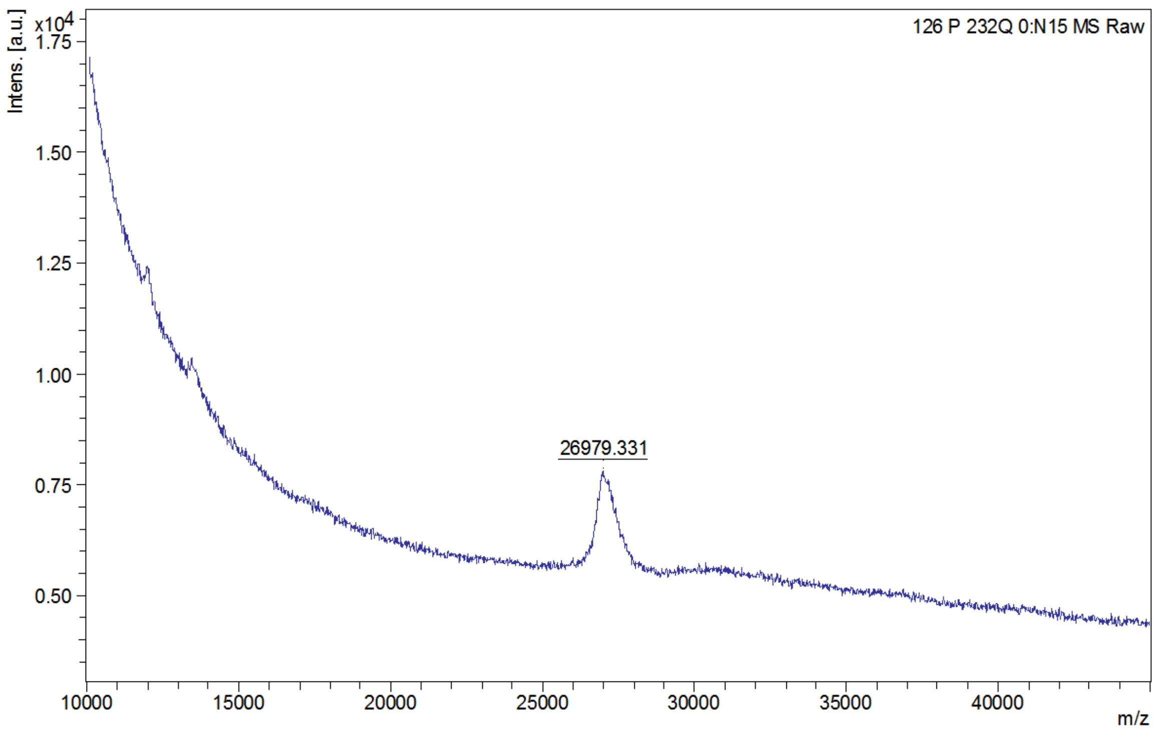

126 P 232Q 0:N15 MS Raw
26979.331
Intens. [a.u.]
x10^4
1.75
1.50
1.25
1.00
0.75
0.50
10000
15000
20000
25000
30000
35000
40000
m/z

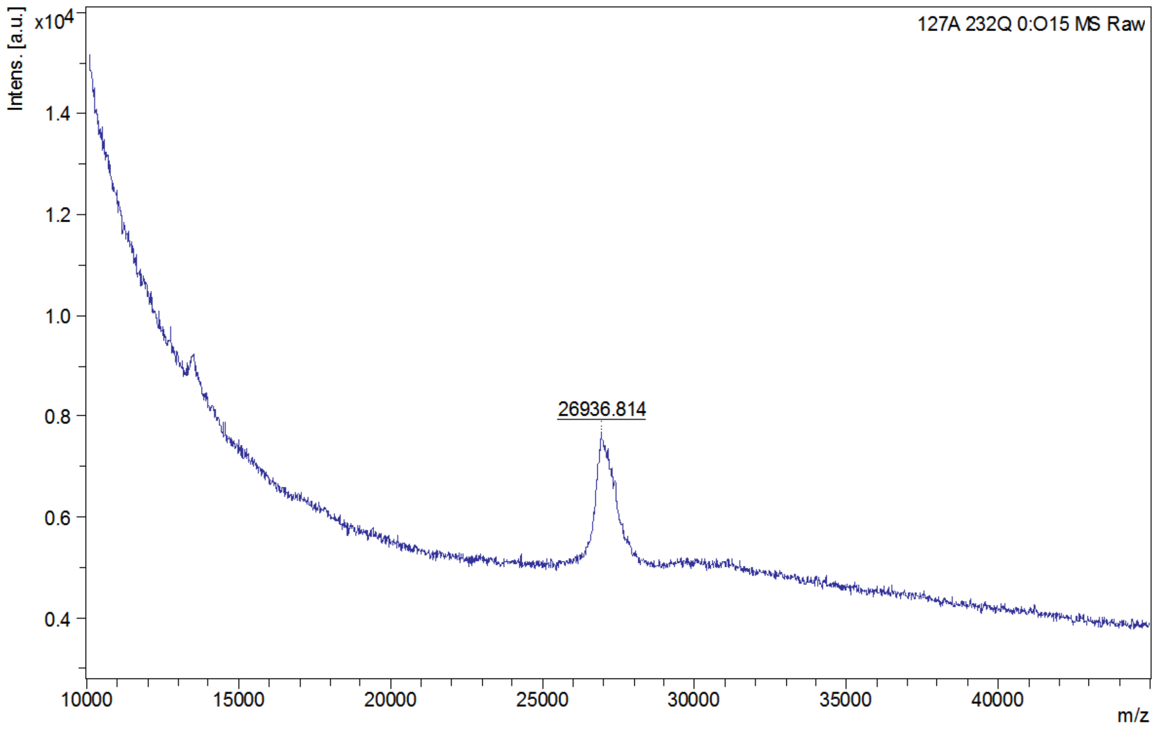

127A 232Q 0:O15 MS Raw
26936.814
Intens. [a.u.]
x10^4
1.4
1.2
1.0
0.8
0.6
0.4
10000
15000
20000
25000
30000
35000
40000
m/z

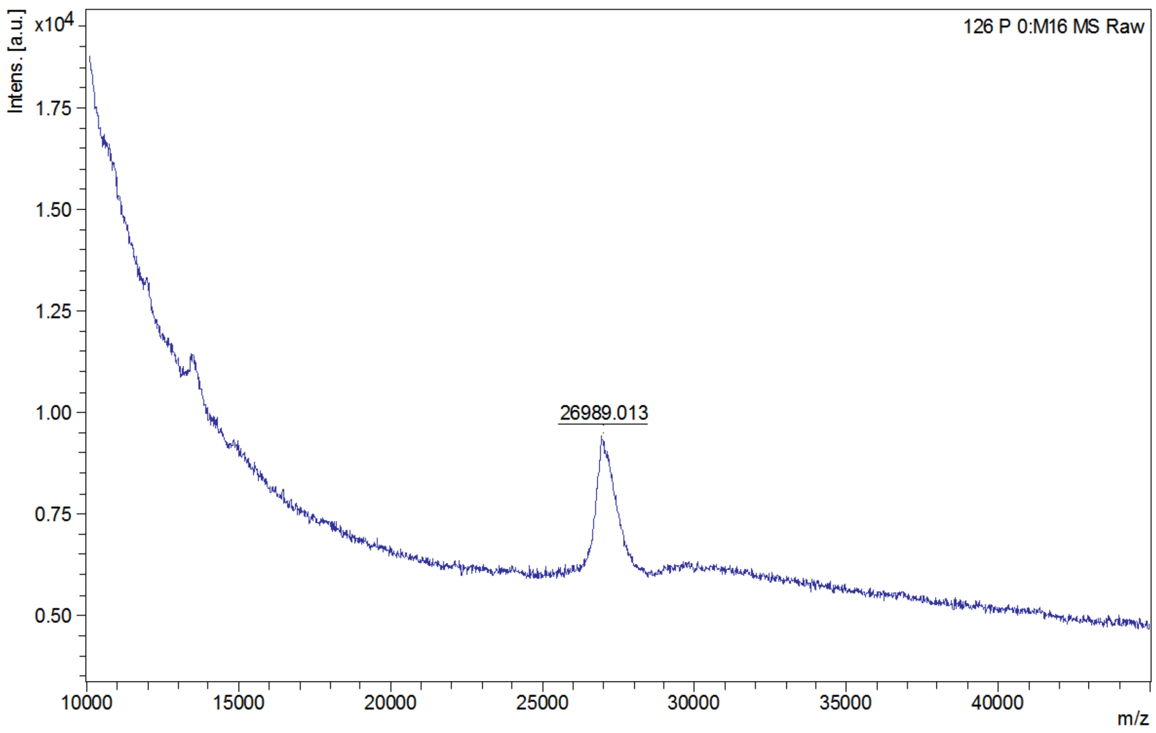

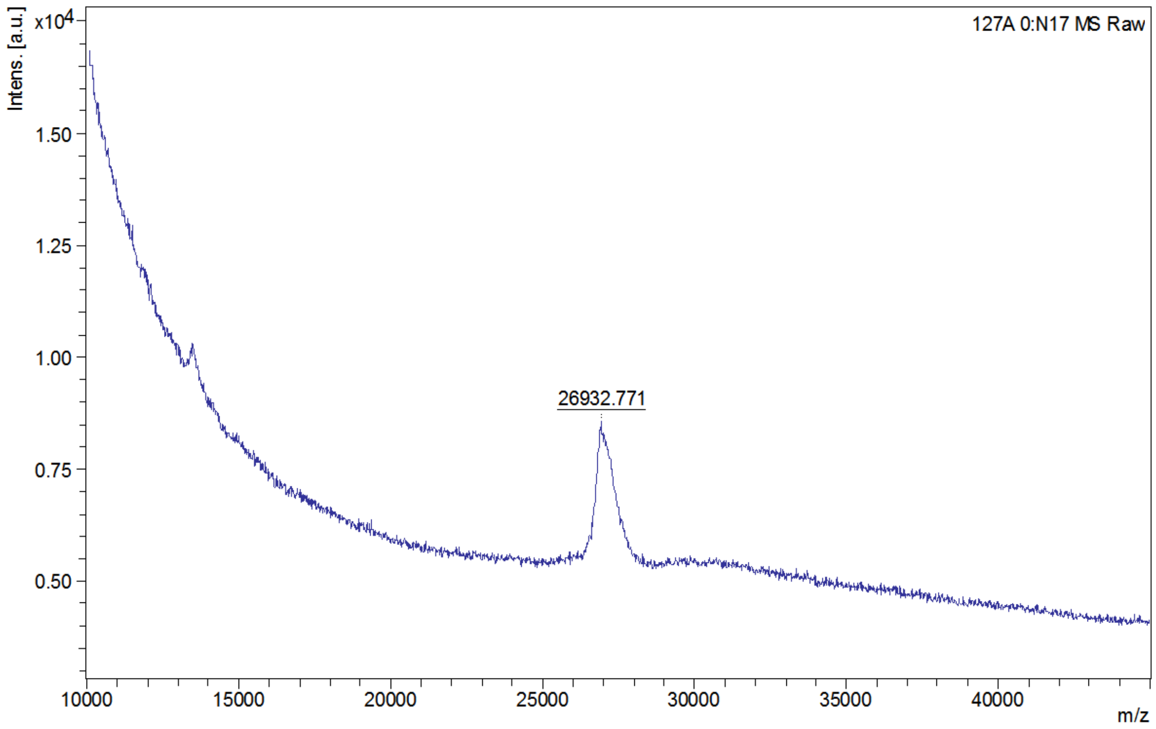

Supplementary Figure 2. The MS results of Lys, 125Q, 232Q, 125232Q, 126P232Q, 127A232Q, 126P, 127A.

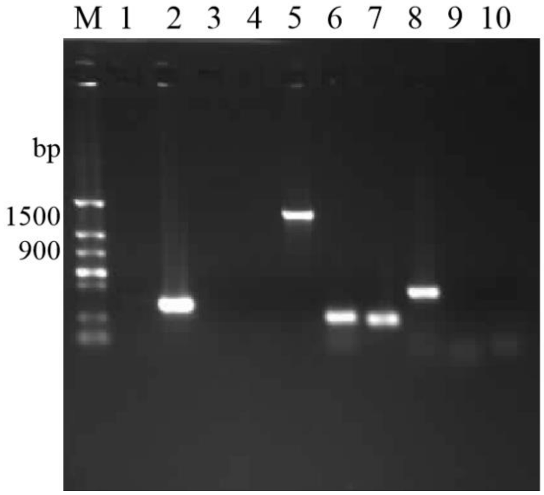

Supplementary Figure 3. Detection of *S. aureus* CVCC 546 virulence genes. M: DNA lander Marker II; Line 1~10: *mecA*; *pvl*; *hla*; *clfA*; *nuc*; *sea*; *psm*-*mec*; *cna*; *fnbpA*; *tsst*-*1*, respectively.

Supplementary Table 1 The selectivity index (SI) values

rLys/Lys	IC_{50} (μg/mL)	MIC (μg/mL) (Mean *S. aureus*)	SI
127A	432.2	4.27	101.2
127A232Q	7899	5.04	1567.3
Lys	18451	6.03	3059.9
C-Lys	4271	20.98	203.6
Amp	3669	0.49	7487.8
